# Development of a conceptual framework to understand the stakeholder’s perspectives on needs and readiness of rural tele-practice for childhood communication disorders

**DOI:** 10.12688/wellcomeopenres.20977.1

**Published:** 2024-05-08

**Authors:** Neethi Jesudass, Vidya Ramkumar, Shuba Kumar, Lakshmi Venkatesh

**Affiliations:** 1 Department of Audiology, Sri Ramachandra Institute of Higher Education and Research (Deemed to be University), Chennai, Tamil Nadu, 600116, India; 2 SAMARTH, Chennai, Tamil Nadu, 600004, India; 3 Department of Speech Language Pathology, Sri Ramachandra Institute of Higher Education and Research (Deemed to be University), Chennai, Tamil Nadu, 600116, India

**Keywords:** Conceptual framework, needs, readiness, rural practice, tele-practice, focus group discussions, semi-structured interviews, qualitative studies, tele-practice, telemedicine

## Abstract

**Background:**

Tele-practice promotes universal and equitable access to quality health services and emerged as an alternative to overcome physical barriers to intervention access in the 90s. There has been a steady increase in adoption since then, and during the COVID-19 pandemic, there was a surge in online modes of healthcare service delivery. Yet, tele-practice adoption and utilization in rural and remote areas are not spontaneous. Therefore, as a first step, prior to the implementation of a comprehensive tele-practice model, a baseline situational analysis was undertaken to assess the needs and readiness of parents of children with disabilities and different cadres of health care providers towards accepting tele-practice services in their settings. This paper describes the process of development of the conceptual framework that guided the baseline needs and readiness assessment (situational analysis).

**Methods:**

The Bowen's feasibility framework served as the primary framework to evaluate the feasibility outcomes of the implementation. Therefore, this framework also guided the baseline situational analysis. For specificity of the framework to tele-practice, several telemedicine planning frameworks relevant for low- and middle-income countries were reviewed to identify and map suitable constructs and attributes to the Bowen’s constructs. A description of the framework selection process and a review of each of the selected telemedicine frameworks are provided.

**Results:**

The constructs and attributes from this conceptual framework were used to develop the guides for focus group discussions (FGDs) and semi-structured interviews (SSIs). The guides were prepared separately for each stakeholder group.

**Conclusions:**

The developed framework facilitated the assessment of needs and readiness suited to the context and among various stakeholders involved in the proposed implementation of the comprehensive model of tele-practice for childhood communication disorders in rural communities.

## Plain Language Summary

This study describes the development of a conceptual framework for assessing the needs and readiness of parents of children with disabilities and different cadres of health care providers regarding their acceptance of tele-practice services in their settings. This baseline situational analysis is an initial step prior to the implementation of a comprehensive tele-practice model for the identification and rehabilitation of children with hearing and speech-language disorders within the public-health system of a rural district in Southern India.

## Introduction

Tele-practice promotes universal and equitable access to quality health services. Digital health can contribute to the sustainability and efficiency of health systems, allowing them to provide affordable, equitable, and high-quality care ( World Health Organization, 2021). Tele-practice emerged in health care sectors to overcome physical barriers to intervention access in the 90s ( Hodge *et al.,* 2019; Law *et al.,* 2021). There has been a steady increase in adoption since then, and during the COVID-19 pandemic there was a surge in online modes of healthcare service delivery ( Lamash *et al.,* 2023; Law *et al.,* 2021; Leung *et al.,* 2023; Shahouzaie & Gholamiyan Arefi, 2022). Therefore, tele-practice is now considered a more mainstream model of health care provision.

Tele-practice has been used to address service delivery gaps in childhood disability ( Akulwar-Tajane & Bhatt, 2021; Camden *et al.,* 2020; Ogourtsova *et al.,* 2023) and more specifically to support rehabilitation of speech, language and hearing disorders ( Dimer *et al.,* 2020; Ebrahimi *et al.,* 2024; Eslami Jahromi *et al.,* 2022). However, most of the tele-practice in audiology and speech language pathology seems to be implemented through funded research ( Ramkumar *et al.,* 2023; Shankar *et al.,* 2022) in high- income countries (HICs) and predominantly in urban settings. But the need is maximum for those living in rural communities who rely largely on public health services ( Roy, 2023; Tulimiero *et al.,* 2021). This is truer for low- and middle-income countries (LMICs). Yet, tele-practice adoption and utilization in rural and remote areas has not been easy owing to reluctance of clinicians in rural areas and rural patients to adopt tele-practice ( Banbury *et al.,* 2023).

The successful implementation of tele-practice services in the public and/or private sectors in rural areas requires careful planning through assessment of the needs of a population and their readiness for availing these services. This is best done using a well thought through conceptual framework that “defines the current state of knowledge (based on literature review), identifies gaps in the understanding of a phenomenon or problem, and provides the methodological basis for the research work” ( Varpio *et al.,* 2020).

A funded project was initiated to assess the feasibility of implementing a comprehensive tele-practice model for the identification and rehabilitation of children with hearing and speech-language disorders in rural communities in Southern India. Therefore, as a first step, prior to implementation, a baseline situational analysis was undertaken to assess the needs and readiness of parents of children with disabilities and different cadres of health care providers towards accepting tele-practice services in their settings. The Bowen's feasibility framework ( Bowen *et al.,* 2009) was selected as the primary framework to evaluate the feasibility outcomes of the implementation. Therefore, this framework also guided the baseline situational analysis. However, for specificity of the framework to tele-practice, several telemedicine planning frameworks were reviewed to identify and map suitable constructs and attributes to the Bowen’s constructs.

This paper describes this process of development and the finalized conceptual framework that guided the baseline needs and readiness assessment (situational analysis).

## Methods

Ethical approval was obtained from Institutional Ethics Committee of Sri Ramachandra Institute of Higher Education and Research (DU) on 25.11.2019 (IEC-NI/19/NOV/71/90).

The COnsolidated Criteria for REporting Qualitative Research (COREQ) Checklist ( Tong *et al.,* 2007) was used to develop and report the domains of study team and reflexivity, and study design (checklist available as *Extended data* ( Ramkumar, 2024)).

The Bowen’s feasibility framework and several telemedicine frameworks applicable for LMICs settings were reviewed. A description of the framework selection process and a review of each of the selected telemedicine frameworks are provided below.

### Review of literature and selection of telemedicine framework

Using search engines and keywords (telemedicine planning or tele-practice, planning, readiness assessment, needs, and framework), a literature search was conducted by the first and second authors (females, audiologist and speech language pathologists). Both of these authors are already trained in qualitative research methods. The literature search led to 22 articles on telemedicine planning. Three experts reviewed these 22 articles and excluded those that did not pertain to LMICs or irrelevant to rural context. Any discrepancies were resolved based on consensus. This resulted in a final set of seven articles that were considered for the mapping process. The summary of the selected telemedicine frameworks is provided in Table 1.

**Table 1.** Description of the selected telemedicine frameworks.

Author, Year and Country	Aim	Method	Constructs
Kiberu *et al.*, 2021, Uganda	Development of an evidence-based e-health readiness assessment framework	Literature review and questionnaire development	Political Readiness Economic Readiness Sociocultural Readiness Technology Readiness Environmental Readiness Legal and Regulatory Readiness
Addotey-Delove *et al.,* 2020, South Africa	Development of a framework for patients’ perspectives on m-health adoption factors in the developing world	Structured literature search and review	Cost and Ownership User Characteristics Language and Literacy Infrastructure Collaboration and Funding Governance, system utility
Kiberu *et al.*, 2019, Uganda	Assessment of core, e-learning, clinical and technology readiness to integrate telemedicine at public health facilities in Uganda	Mixed methods using questionnaire and focus group discussion	Core Readiness E-learning Readiness Clinical Readiness Technology readiness
Mauco *et al.*, 2018, Botswana	Critical analysis of e-health readiness assessment frameworks: suitability for application in developing countries	Literature review	Organisational readiness Technological/ Infrastructural readiness Healthcare provider readiness Engagement readiness Societal readiness Core readiness Government readiness Public/ Patient readiness
AlDossary *et al.*, 2017, Australia	Development of a planning framework for telemedicine services based on needs assessment	Mixed methods using questionnaire, focus groups and geographic information analysis	Availability and expressed needs, Accessibility, Perception and affordability
Kgasi & Kalema, 2014, South Africa	Development of framework for e-health readiness assessment	Questionnaire	Core Readiness, Structural Readiness, Effort Expectance Performance, Expectance Engagement Readiness Societal Readiness
Jennett *et al.*, 2003, Canada	Development of readiness model for telehealth	Key informant interviews, awareness sessions, focus groups, and face-to-face interviews	Core readiness Engagement readiness Structural readiness Non-readiness

Three experts with experience in tele-practice reviewed the constructs of the finalized seven articles on telemedicine planning. The identified concerns were subsequently addressed, and the conceptual framework was finalised.

### Mapping the attributes of telemedicine framework to the Bowen’s feasibility framework

The primary framework to study feasibility outcomes was the Bowen's feasibility framework ( Bowen *et al.,* 2009) which includes the constructs of acceptability, demand, implementation, practicability, adaptation, integration, expansion, and limited efficacy. While the construct of demand was chosen to understand the demands or needs of tele-practice, the constructs of acceptability, integration, and practicality were chosen to understand the readiness of tele-practice. The study team visited the research locations to gain knowledge about the current health services, systems, infrastructure available and various cadres of health care providers in these communities.

The constructs were reviewed and included in an iterative manner guided and influenced by the understanding of the research locations. Using the constructs identified following our telemedicine frameworks literature review, three experts mapped these to the constructs of Bowens feasibility framework to explore the needs and readiness for tele-practice in rural areas for childhood communication disorders ( Table 2). All these attributes were selected based on the study's research objectives.

**Table 2.** A conceptual framework to study the needs and readiness for tele-practice in rural areas for childhood communication disorders.

S.No.	Constructs included from Bowen's feasibility framework	Constructs included from reviewed Telemedicine frameworks	Attributes that exemplifies the constructs specific to the research objectives
1	Demand/Need	Availability, accessibility, expressed needs	Availability of diagnostic and rehabilitation facilities
			Accessibility of diagnostic and rehabilitation facilities
			Barriers and challenges in seeking health care (transport, timing, cost)
			Satisfaction with the quality of health care
2	Acceptability	Acceptance of service	Use of communication links between stakeholders
			Participation
			Attitudes of stakeholders
			Customs and beliefs with respect to disabilities or health care services
		Comfort with technologies	Comfort with the use of mobile phone-based screening
			Comfort with hearing and speech-language screening by grass root workers
3	Integration	Organizational readiness	Policy will
			Willingness to adapt health systems
			Infrastructure availability/capacity
		Clinical skills	Training needs
			Learning readiness
		Time	Time management by providers
		Perceived fit	
4	Practicality	Technical aspects	Training needs, learning readiness
		Economic	Caregivers and healthcare providers perspectives
		Affordability	Caregivers’ perspectives
			Healthcare provider perspectives
			Technical

## Results

This conceptual framework was used to obtain the perspectives of various stakeholders involved in the study to assess their needs and readiness towards accepting tele-practice services in their settings. The stakeholders were health care providers (HCPs) and parents in two rural districts in the state of Tamil Nadu with similar contexts (Ariyalur and Perambalur). The HCPs included the District Differently Abled Welfare (DDAW) officers, multi-purpose rehabilitation therapist, Audiologist and speech-language pathologists (ASLPs) working in public-sector services, special educators in early intervention centres (EICs), Early Intervention Center (EIC) – In-charge, SSA Special educators, government pre-school teachers ( *anganwadi workers*) and Village Health Nurses (VHNs). Parents of children with disabilities were also included.

The conceptual framework were used to develop the guides for focus group discussions (FGDs) and semi structured interviews (SSIs) (available as *Extended data* ( Ramkumar, 2024)). The guides were prepared separately for each stakeholder group. We followed the five phases for the development of the SSI guide that were provided by Kallio *et al*. (2016): identifying the prerequisites for using semi-structured interviews; retrieving and applying prior knowledge; formulating the preliminary semi-structured interview guide; pilot testing the guide; and presenting the complete guide. We followed the same five phases for the development of the FGD guide as well. 

The guides were further enhanced and improved by integrating the insights obtained from each focus group discussion and interview, thereby enabling a deeper understanding. Prior to data collection, two specialists with expertise in qualitative research and tele-practice conducted a further review of the guides to ensure that they were concise, easy to comprehend, and pertinent to the research objectives. The main questions were followed by probe questions to ensure that the perspectives obtained were pertinent to the research question. Two experts in the field validated the guides, and based on their suggestions, modifications were made to the guides. The guide was then tailored to the needs of each stakeholder group. Prior to the data collection, a pilot test was conducted with the guide.

 Interviews with a semi-structured method were selected to collect individual responses to the research question. If any stakeholder group had less than five members, an SSIs was conducted with the participant's consent. The guides were used to understand perceptions of the health care providers on their training needs, infrastructure availability, time management between administrative and clinical work and challenges in providing consistent diagnostic and rehabilitation services will be explored. Their perceptions of barriers and facilitators to tele-practice implementation and their readiness for adopting it were explored. More specifically, SSI guides were developed for the DDAW officers, multi-purpose rehabilitation therapist ASLPs, special educators and EIC incharges, FGDs were carried out with SSA Special educators, *anganwadi workers* and VHNs as well as with parents of children with disabilities. A separate guide was developed to understand caregiver perceptions regarding the availability and accessibility of diagnostic and rehabilitation facilities, barriers and challenges encountered in seeking care, and satisfaction with quality of care, including cost-related issues and their readiness to accept tele-practice.

## Conclusion

A context and content specific needs and readiness framework was developed to guide the implementation of tele-practice for childhood communication disorders in rural communities.

## Limitations

The conceptual framework was only implemented within a limited geographic area, specifically confined to a few districts in the southern region. It will be worthwhile to apply this framework to other regions in India and other low- and middle- income countries.

## Ethics and consent

Ethical approval was obtained from Institutional Ethics Committee of Sri Ramachandra Institute of Higher Education and Research (DU) on 25.11.2019 (IEC-NI/19/NOV/71/90). The informed consent forms were signed by participants prior to the data collection.

## Data Availability

The data for this article consists of bibliographic references, which are included in the References section, and are outlined in Table 1 and Table 2. Open Science Framework: Method Manuscript. https://doi.org/10.17605/OSF.IO/4P3M2 ( Ramkumar, 2024). This project contains the following extended data: COREQ checklist Guides for FGD and SSI developed using the conceptual framework for health care providers Guides for FGD and SSI developed using the conceptual framework for parents Data are available under the terms of the Creative Commons Attribution 4.0 International license (CC-BY 4.0).
