# Supplementary material for: Development of a conceptual framework to understand the stakeholder’s perspectives on needs and readiness of rural tele-practice for childhood communication disorders
Source: Wellcome Open Res. 2024 May 8;9:239. [Version 1] doi: 10.12688/wellcomeopenres.20977.1 (PMC11362737; doi:10.12688/wellcomeopenres.20977.1)
Supplement: Peer review history

# Reviewer report: Reviewer response for version 1

**DOI:** 10.21956/wellcomeopenres.23210.r88014

**Published:** 2024-09-06

**Authors:** Kate Bridgman

**Affiliations:** 1 La Trobe University, Melbourne, Victoria, Australia

Thank you for the opportunity to review this article titled ‘Development of a conceptual framework to understand the stakeholder’s perspectives on needs and readiness or rural tele-practice for childhood communication disorders’. The authors are to be commended for the detail of this study – first conducting a review to select suitable frameworks to then inform focus group interviews.

**The following comments are offered to refine specific details to increase clarity for a first-time reader:**

1. **Terminology** – the population is referred to differently throughout the manuscript, for e.g. childhood communication disorders (titled), children with disabilities (abstract), children with hearing and speech-language disorders (plain English summary and introduction). For clarity, it would be best to use title ‘childhood communication disorders’ throughout.
2. **History of telepractice** – the authors reports that telepractice emerged in the 90s, however use and development of telepractice/telehealth predates the 1990s (for e.g. The Evolution of Telehealth: Where Have We Been and Where Are We Going? - The Role of Telehealth in an Evolving Health Care Environment - NCBI Bookshelf (nih.gov))
3. **References ** - introduction paragraph 3 – a reference would strengthen the statement ‘ *The successful implementation of tele-practice services in the public and/or private sectors in rural areas requires careful planning through assessment of the needs of a population and their readiness for availing these services.’*
4. **Details of search **–  are the exact search terms listed? Typically the terms including truncations, wildcards and Boolean operators are listed, as are the exact databases that are used.
5. **Constructs – **Table 1 is well presented and represents a summary of each framework, an additional column explaining the construct of definition may help readers with new concepts – for e.g. what is meant by ‘technology readiness’ and ‘E-learning readiness’, and how might these differ? What is meant by, or an example of ‘effort expectance’?. This could also be included for Table 2 – for e.g. what is meant by ‘policy will’?

Is the rationale for developing the new method (or application) clearly explained?

Yes

Is the description of the method technically sound?

Yes

Are the conclusions about the method and its performance adequately supported by the findings presented in the article?

Yes

If any results are presented, are all the source data underlying the results available to ensure full reproducibility?

Partly

Are sufficient details provided to allow replication of the method development and its use by others?

Partly

Reviewer Expertise:

Telehealth, implementation science.

I confirm that I have read this submission and believe that I have an appropriate level of expertise to confirm that it is of an acceptable scientific standard, however I have significant reservations, as outlined above.

## References

[1] Board on Health Care Services; Institute of Medicine. The Role of Telehealth in an Evolving Health Care Environment: Workshop Summary. Washington (DC): National Academies Press (US).2012 Nov 20. 3, The Evolution of Telehealth: Where Have We Been and Where Are We Going? Available from: https://www.ncbi.nlm.nih.gov/books/NBK207141/;24901186

# Reviewer report: Reviewer response for version 1

**DOI:** 10.21956/wellcomeopenres.23210.r88016

**Published:** 2024-08-29

**Authors:** Tanvi Sanghavi

**Affiliations:** 1 All India Institute of Speech and Hearing, Mysore, India

The idea of the article is relevant and meaningful although the title can be simplified and kept short. The experience of audiologists and speech language pathologists is also worth mentioning. Please mention if they have taken any training for providing teletherapy series. As future research, the authors can develop a model to make teletherapy a viable option and find out the barriers (if any) for opting the tele mode.

Is the rationale for developing the new method (or application) clearly explained?

Yes

Is the description of the method technically sound?

Yes

Are the conclusions about the method and its performance adequately supported by the findings presented in the article?

Yes

If any results are presented, are all the source data underlying the results available to ensure full reproducibility?

No source data required

Are sufficient details provided to allow replication of the method development and its use by others?

Yes

Reviewer Expertise:

Telepractice, autism, speech language pathology, dysphagia

I confirm that I have read this submission and believe that I have an appropriate level of expertise to confirm that it is of an acceptable scientific standard.

# Reviewer report: Reviewer response for version 1

**DOI:** 10.21956/wellcomeopenres.23210.r92846

**Published:** 2024-08-14

**Authors:** Mary Wickenden

**Affiliations:** 1 Institute of Development Studies, Brighton, England, UK

Thanks for the opportunity to review this interesting piece which presents the development of a  conceptual framework for a needs assessment for a new tele-practice to provide speech and language therapy for children with communication impairments in rural South India. It draws on a number of previously produced frameworks to develop one suitable for this purpose.

 It is generally well written, clear, succinct and easy to follow.  It is a systematic description of the process.

 There is very little need for change, but I have listed a few places where the expression could be clearer or more grammatical, see below:

**In results section:**

- End of 1st para- 'Parents of children with disabilities were also included'  This makes them sound like an afterthought, whereas surely they are probably the most important group of respondents?  Adjust the wording maybe?.
- 2nd para- The conceptual framework was used.
- ‘Interviews with a semi-structured method’  - adjust to >  'Interviews using a semi-structured'.
- 'an SSIs was conducted', adjust to > 'an interview was conducted'.
- 'The guides were used to understand perceptions of the health care providers on their training needs, infrastructure availability, time management between administrative and clinical work and challenges in providing consistent diagnostic and rehabilitation services will be explored'. Need to adjust grammar of these sentences.
- 'their readiness for adopting it were explored' adjust to > readiness to adopt it.
- Last few sentences of last paragraph in Results are slightly confusingly worded.  You’ve already said that you specifically designed the topic guide for each group, including parents and then you mention caregivers. Need to adjust this paragraph for consistency.  However, giving the example of the kinds of things you planned to ask parents/caregivers is good though, so retain this in your rephrase.

**Additionally **- I would have been interested in some mention of the possibility of asking children themselves what they would think about a tele therapy approach. You clearly weren't thinking of doing that but the possibility deserves a mention!

Is the rationale for developing the new method (or application) clearly explained?

Yes

Is the description of the method technically sound?

Yes

Are the conclusions about the method and its performance adequately supported by the findings presented in the article?

Yes

If any results are presented, are all the source data underlying the results available to ensure full reproducibility?

Partly

Are sufficient details provided to allow replication of the method development and its use by others?

Yes

Reviewer Expertise:

Speech and language therapist and anthropologist, with research interests in disability in the Global South. Particularly  developing and using qualitative, participatory , disability inclusive projects, asking children and families about their experiences and perspectives. Working with disabled children and adults as peer researchers and active participants. Geographical foci in India, Sri Lanka, Bangladesh and also in East Africa.

I confirm that I have read this submission and believe that I have an appropriate level of expertise to confirm that it is of an acceptable scientific standard.
